# Protein‐Driven Copper Redox Regulation: Uncovering the Role of Disulphide Bonds and Allosteric Modulation

**DOI:** 10.1002/anie.202519673

**Published:** 2026-02-27

**Authors:** Rebecca Sternke‐Hoffmann, Chang Liu, Xue Wang, Hegne Pupart, Xun Sun, Jan Gui‐Hyon Dreiser, Peep Palumaa, Qinghua Liao, Matthias Krack, Jinghui Luo

**Affiliations:** ^1^ PSI Center for Life Sciences Villigen PSI Switzerland; ^2^ Department of Chemistry and Biotechnology Tallinn University of Technology Tallinn Estonia; ^3^ PSI Center for Photon Science Villigen PSI Switzerland; ^4^ Departament de Química Inorgànica i Orgànica (Secció de Química, (Orgànica) Institut de Química Teòrica i Computacional (IQTCUB) Universitat de Barcelona Barcelona Spain; ^5^ PSI Center for Scientific Computing, Theory and Data Villigen PSI Switzerland

**Keywords:** Cu(II)/Cu(I) Reduction, disulphide bond, HSA, plasma protein redox biology, SAXS

## Abstract

Copper plays essential roles in enzymatic activity, redox reactions, and cellular signalling but becomes toxic when redox homeostasis is disrupted. While Cu(II) reduction is commonly attributed to unfolded or amyloid proteins, here we show that the well‐folded plasma protein human serum albumin (HSA) intrinsically reduces Cu(II) to Cu(I) in the absence of external reductants. Using x‐ray absorption spectroscopy (XAS), small‐angle x‐ray scattering (SAXS), and circular dichroism (CD), we propose a redox mechanism involving the disulphide bond Cys392‐Cys438 in domain III of HSA. Cu binding at the high‐affinity ATCUN (amino‐terminal copper and nickel binding site) motif might trigger conformational changes that expose this disulphide bond, enabling thiol‐mediated electron transfer and Cu(I) formation. Chelation with tetrathiomolybdate (TTM) impairs this reduction by restricting access to the reactive disulphide site. Comparative analysis with other globular proteins reveals that Cu reduction requires both accessible disulphide motifs and a native folded structure. Simulations and spectroscopy of SOD1 (Superoxide Dismutase 1) confirm that disulphide cleavage enhances Cu‐thiolate interaction, supporting a generalisable two‐site redox mechanism. These findings reveal a previously unrecognised mode of protein‐mediated copper reduction and suggest broader physiological roles for disulphide‐regulated redox switching in metal homeostasis.

## Graphical abstract

Copper binding triggers structural changes that expose a reactive disulphide bridge, enabling intrinsic Cu(II) reduction in folded proteins. SAXS, XAS, and QM/MM simulations reveal how globular proteins such as HSA and SOD1 employ disulphide‐mediated dynamics to modulate site accessibility and control copper redox chemistry.

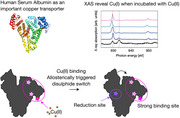

## 1 Introduction

Living organisms depend on various transition metals for proper functioning, and copper is a prime example. Copper plays a crucial role in enzymatic catalysis, intracellular signalling, and redox balance [1].

Copper's redox versatility enables it to participate in single‐electron transfers, primarily existing in two oxidation states: Cu(I) and Cu(II) [2]. Although this property is vital for enzyme functions, it also contributes to oxidative stress through the formation of hydroxyl radicals from the Haber‐Weiss reaction, potentially damaging biomolecules [3]. To mitigate such risks, organisms have developed a sophisticated regulatory system that collectively maintains copper homeostasis through specific proteins [4, 5].

Intracellularly, Cu(I) predominates in the reducing environment maintained by glutathione (GSH) and cysteine [6]. In contrast, Cu(II) is stabilised in extracellular fluids, especially within high‐affinity protein‐binding sites [7]. The redox conversion between these two states is central to copper's biological activity and toxicity. Cysteine residues, in particular, play key roles in both binding and redox conversion, due to their thiol chemistry [8, 9, 10]. Copper coordination preferences vary by oxidation state. Cu(I), a $d^{10}$ cation, exhibits minimal stabilisation energy of the ligand field and prefers coordination with soft ligands such as His, Cys and Met [11, 12]. Cu(II), a $d^{9}$ cation, adopts square planar, square pyramidal, or axially distorted octahedral geometries due to Jahn–Teller distortion [13]. Ligand preferences align with the theory of hard and soft acids and bases (HSAB), as demonstrated by cyclen‐based ligands that removed Cu(II) from Alzheimer's amyloid‐β (Aβ), while sulphur‐containing analogues preferentially bind Cu(I) [14, 15].

Previous studies have primarily linked Cu(II) reduction to amyloid and intrinsically disordered proteins, implicating histidine, tryptophan, and cysteine residues in redox activity [16, 17, 18, 19, 20, 21, 22, 23, 24, 25]. Amyloid‐β (Aβ), for instance, can reduce Cu(II) to Cu(I) [24], which may exacerbate oxidative stress and contribute to neurotoxicity. Some studies propose that amyloid proteins facilitate the transport of copper across cellular membranes, but pathological aggregation may exacerbate Cu(II) reduction and oxidative stress [26]. However, the physiological relevance of this process remains debated, as Aβ concentrations in vivo are far lower than those for plasma proteins such as human serum albumin (HSA) [27, 28]. In the bloodstream, copper is primarily bound to metalloproteins, such as ceruloplasmin, while a small labile fraction interacts with proteins, mainly HSA, amino acids, and peptides [29, 30, 31].

Disruptions in copper regulation contribute to disease pathogenesis. Wilson's disease (WD) and Menke's disease (MD) arise from mutations in the copper transporters ATP7B and ATP7A, respectively, leading to copper mislocalisation [32]. In WD, excessive copper accumulation damages the liver and brain, while in MD, copper deficiency disrupts essential enzymatic functions. Furthermore, Amyotrophic Lateral Sclerosis (ALS) is linked to mutations in the metalloprotein Cu, Zn‐superoxide dismutase (SOD1), which impair copper binding, contribute to protein aggregation and thereby hinder the neutralisation of free radicals [32]. Understanding copper homeostasis is also critical for therapeutic development, such as optimising metal chelators like tetrathiomolybdate (TTM).

Here, we investigate whether well‐folded globular proteins also possess intrinsic Cu(II)‐reducing activity in the absence of external reductants. We focus on HSA, the most abundant plasma protein and a major copper carrier in the bloodstream [33]. HSA contains a high‐affinity Cu(II)‐binding site known as the ATCUN (Amino‐Terminal Cu‐ and Ni‐binding) motif, as well as additional metal‐binding regions and 17 disulphide bonds [34]. HSA and its ATCUN motif DAH exhibit a strong Cu(II) affinity with $K_{d}$ of 9.55 x 10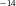 M and 2.00 × 10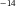 M, respectively [35, 36]. Beyond HSA, ATCUN motifs are found in diverse proteins across biological compartments [37, 38]. While previous work suggested that Cu(I) formation on HSA requires external reductants like ascorbate, we now demonstrate that HSA alone can reduce Cu(II) to Cu(I) under physiological conditions.

To elucidate this unexpected redox behaviour, we combine x‐ray absorption spectroscopy (XAS) and small‐angle x‐ray scattering (SAXS) with other biophysical methods, and compare the redox efficiency between HSA and three different ATCUN motif peptides: DAH (from HSA), DTHFPI (from hepcidin)[39, 40], and MEHFPGP (from semax)[41, 42]. While previous studies suggest that these peptides display strong Cu(II) affinity [35, 41, 43, 44, 45, 46], direct competition experiments indicate comparable binding strengths, with DAH exhibiting the lowest dissociation constant [36]. We propose a previously unrecognised redox‐active disulphide bond (Cys392‐Cys438) in HSA's domain III distant from the ATCUN motif as the most likely redox‐active disulphide. Cu binding at the ATCUN motif induces allosteric rearrangements that expose this site, facilitating copper reduction via thiol coordination.

To assess the generality of this mechanism, we extend our analysis to other globular proteins (including β‐lactoglobulin, insulin, lysozyme and the metalloprotein SOD1). While only HSA shows strong Cu(II)‐reducing activity in its native fold, SOD1 also reveals comparable disulphide‐gated Cu redox switch upon bond cleavage. These findings suggest that intrinsic redox regulation via conformational gating of disulphide bonds may be a broader property of globular proteins than previously appreciated.

## 2 Results

### 2.1 HSA Reduces Copper via a Mechanism Independent of Its High‐Affinity ATCUN Motif

**FIGURE 1:**
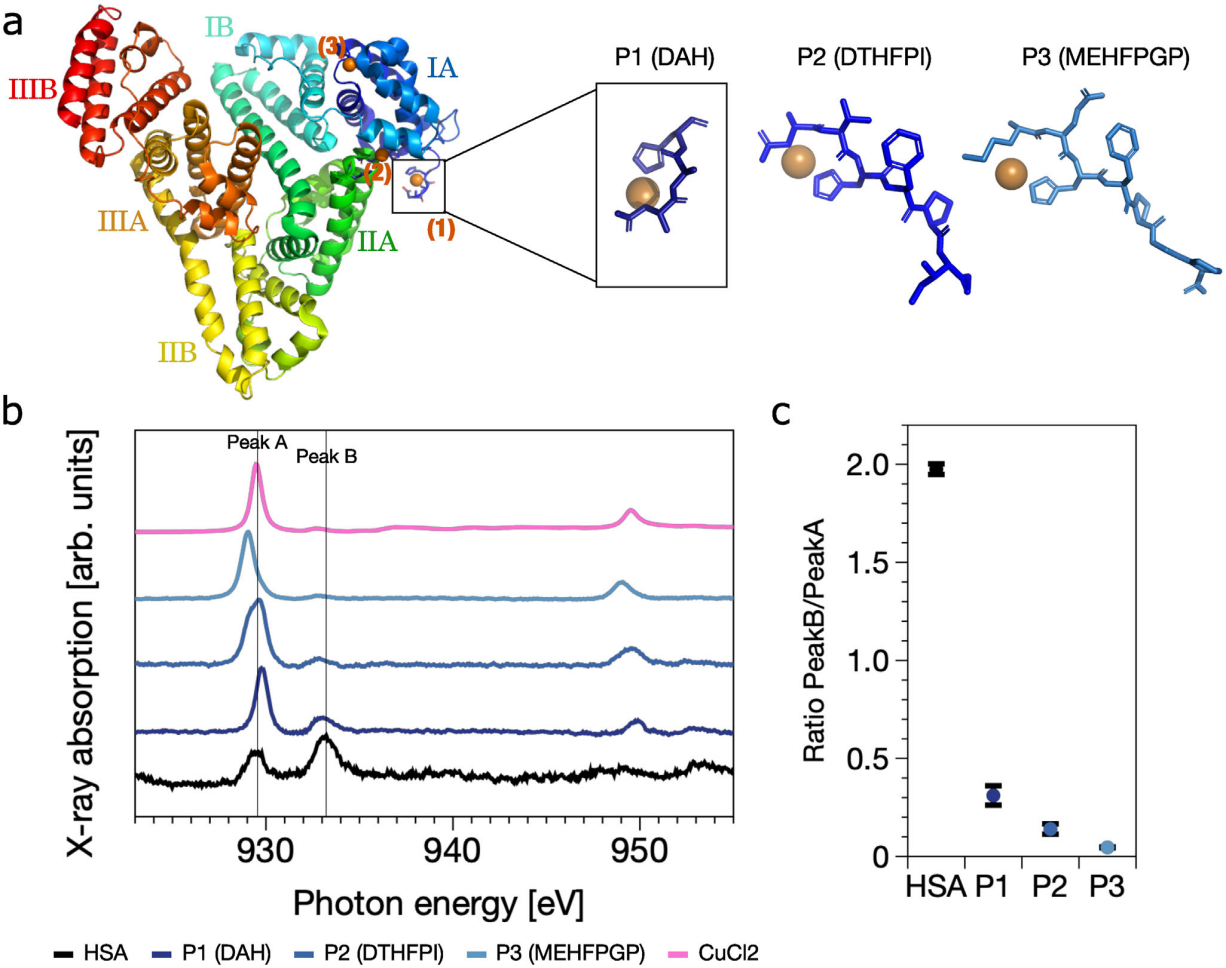
HSA reduces Cu(II) to Cu(I) via mechanism distinct from ATCUN motif. (a) Structural representations of human serum albumin (HSA, PDB ID: 7WLF [48]) and the three ATCUN peptides: DAH (P1), DTHFPI (P2), and MEHFPGP (P3), generated via AlphaFold. In HSA, the primary copper‐binding sites are highlighted in orange and located around domain I. (b) Soft x‐ray absorption spectra recorded at the Cu $L_{2,3}$‐edges for HSA (black), ATCUN motif peptides P1 (dark blue), P2 (blue) and P3 (light blue), incubated with ${\mathrm{CuCl}}_{2}$ (1:2 molar ratio) at pH 7.4. ${\mathrm{CuCl}}_{2}$ alone is shown in pink. Peak A (Cu(II), ∼929.6 eV) and B (Cu(I), ∼933.2 eV) are indicated with vertical lines. (c) Ratio of integrated Peak B to Peak A areas, quantifying the extent of Cu(I) formation. HSA shows a substantially higher Cu(I)/Cu(II) ratio than any ATCUN peptide, suggesting that the ATCUN motif does not drive the observed reduction. Hard x‐ray absorption data at the Cu K‐edge (Figure S1) confirm the presence of Cu(I) in HSA and reveal a distinct coordination geometry compared to ascorbate‐reduced Cu(I), supporting the involvement of a different copper‐binding site.

HSA, a key copper transporter, can bind up to five copper ions [47]. Three primary binding sites have been identified in its N‐terminal domain. These include the ATCUN motif (Asp–Ala–His), a multi‐metal site at the IA‐IIA interface, and a reduced cysteine at position 34, which are indicated on the crystal structure (Figure 1a).

To investigate the role of HSA's high‐affinity ATCUN motif in copper reduction, we compared full‐length HSA with three peptides containing canonical ATCUN motifs—DAH (P1), DTHFPI (P2) and MEHFPGP (P3)—using soft x‐ray absorption spectroscopy (XAS). Upon incubation with ${\mathrm{CuCl}}_{2}$ (1:2 molar ratio (protein:${\mathrm{CuCl}}_{2}$)), the Cu L‐edge spectra of HSA displayed two key features: Peak A (at ∼929.6 eV), characteristic of Cu(II), and Peak B (at ∼933.2 eV), indicative of monovalent Cu(I) (Figure 1b). In contrast, the ATCUN peptides showed only Peak A with minimal or no Peak B signal (Figures 1b,c and S1). This comparison indicated that Cu(II) is not reduced by the ATCUN motif alone and that the reduction observed in HSA occurs through a distinct mechanism. The stronger the binding affinity of copper to an ATCUN peptide, the lower the peptide's ability to reduce the metal, as exemplified by P3. The faint Cu(I) signals detected for the P1 and P2 ATCUN peptides likely originate from more flexible or incomplete coordination geometries, which render these complexes slightly more prone to reduction by nearby residues. Nevertheless, the precise mechanism underlying this behaviour remains to be elucidated.

Hard x‐ray spectroscopy at the Cu K‐edge confirmed the presence of Cu(I) in HSA, and revealed that its coordination geometry differs significantly from the two‐coordinate linear Cu(I) species formed by ascorbate reduction (Figure S2). This suggests that Cu(II) is reduced to Cu(I) at a site outside the canonical high‐affinity binding motifs and adopts a likely four‐coordinate geometry [49, 50].

### 2.2 Chelation Reveal Allosteric Modulation of Copper Reduction in HSA

**FIGURE 2:**
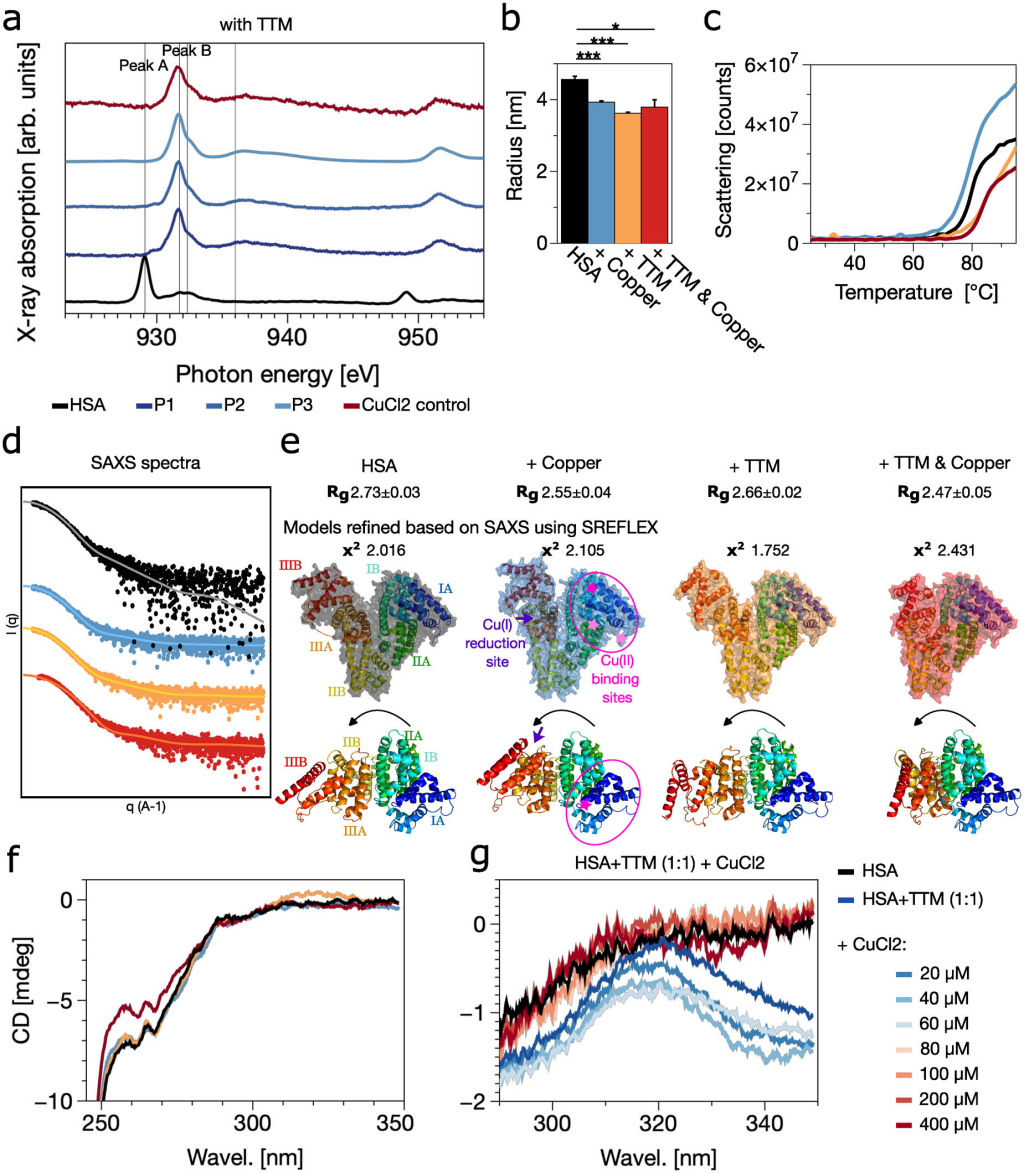
Copper chelation reveals allosteric regulation of HSA for Cu(II) reduction. (a) Soft x‐ray absorption spectra at the Cu $L_{2,3}$‐edges for 100 μM HSA (black) and three ATCUN peptides (DAH (P1 dark blue), DTHFPI (P2, blue) and MEHFPGP (P3, light blue)) incubated with ${\mathrm{CuCl}}_{2}$ (200 μM) and TTM (100 μM) at pH 7.4. While Cu(II) signals are suppressed in peptides (spectra resemble Cu‐TTM control), HSA retains a prominent Cu(II) peak, suggesting TTM interferes with reduction in HSA. (b) Hydrodynamic radius of HSA measured by DLS, pairwise *p*‐values are indicated by asterisk (*p*<0.05 (*), *p*<0.001 (***)). Copper and TTM both lead to compaction. (c) DSF‐derived thermal unfolding profiles show increased thermal stability upon TTM binding, consistent with structural stabilisation. (d) SAXS profiles (black: HSA; blue: +${\mathrm{CuCl}}_{2}$; orange: +TTM; red: +${\mathrm{CuCl}}_{2}$+TTM) with model fits (lighter colours) and (e) SAXS‐derived models (via SREFELX, 4LA0 [53]) show compaction localised to domain IIIA/IIIB (red/orange), while canonical Cu‐binding sites in domain I (marked with pink) remain structurally unchanged. (f) Near‐UV CD spectra reveal changes in aromatic residue environments, especially in Cu‐TTM‐HSA complex. (g) CD signal around 320 nm (indicative of disulphide formation) appears upon TTM binding but diminishes upon Cu titration, suggesting competition between TTM and Cu for redox‐active cysteines.

To explore whether Cu(II) reduction in HSA involves allosteric mechanisms or specific structural regions, we examined the impact of tetrathiomolybdate (TTM), a high‐affinity copper‐binding chelator, on copper redox behaviour and protein conformation. TTM is commonly used in the treatment of Wilson's disease and cancer therapy, as it effectively depletes bioavailable intracellular copper [51, 52]. XAS spectra of Cu‐TTM complexes in solution and with ATCUN peptides resembled Cu(I) (Figure 2a), indicating that TTM stabilises the reduced state, consistent with its strong Cu(I) binding capability. When mixed with ATCUN peptides, TTM led to a nearly complete loss of the Cu(II) signal, suggesting that peptide‐bound Cu is easily accessible for chelation and reduction. The shift from cupric copper in the absence of chelating agent toward cuprous copper in the presence of TTM was particularly evident for peptide P3 (see Figure S2).

However, in HSA, TTM had the opposite effect: Cu(II) was retained, and Cu(I) formation was suppressed. This indicates that TTM interferes with the reduction process, either by limiting Cu mobility, shielding redox‐active sites, or altering HSA's structure. To support this, dynamic light scattering (DLS), differential scanning fluorimetry (DSF), and circular dichroism (CD) measurements confirmed that TTM binding induces structural changes in HSA, compacting and stabilizing the protein (Figure 2b,c). SAXS analysis (see Figure S3) with SREFELX modelling localised these conformational changes in domains IIIA and IIIB, which are distal to the Cu‐binding ATCUN motif in domain I (Figure 2d,e). However, increasing the ${\mathrm{CuCl}}_{2}$ and TTM concentrations (≥1:3) led to destabilisation, aggregation and partially unstructured conformations (as shown in Figures S4–S7). These structural changes were absent in domain I, where the canonical Cu‐binding sites are located, suggesting an allosteric transmission of Cu binding events to more distal redox‐active regions.

Circular dichroism further confirmed local structural rearrangements and perturbations of the disulphide bond upon TTM and copper binding (Figure 2f,g). A slight perturbation in the local environment of phenylalanine and tyrosine residues could be observed within the Cu‐TTM‐HSA complex. TTM and HSA alone induced a signal consistent with disulphide formation (Figure S8), which was abolished by titration of Cu(II), suggesting a dynamic interplay between disulphide status and copper redox activity. In general, allosteric changes induced by TTM might obstruct access to redox‐active disulphide site and thereby inhibiting copper reduction.

These findings support the idea that copper binding initiates allosteric structural rearrangements that may propagate to remote regions containing disulphide bonds.

### 2.3 Disulphide Bond Cys392–Cys438 is a Likely Redox‐Active Site for Cu(II) Reduction in HSA

**FIGURE 3:**
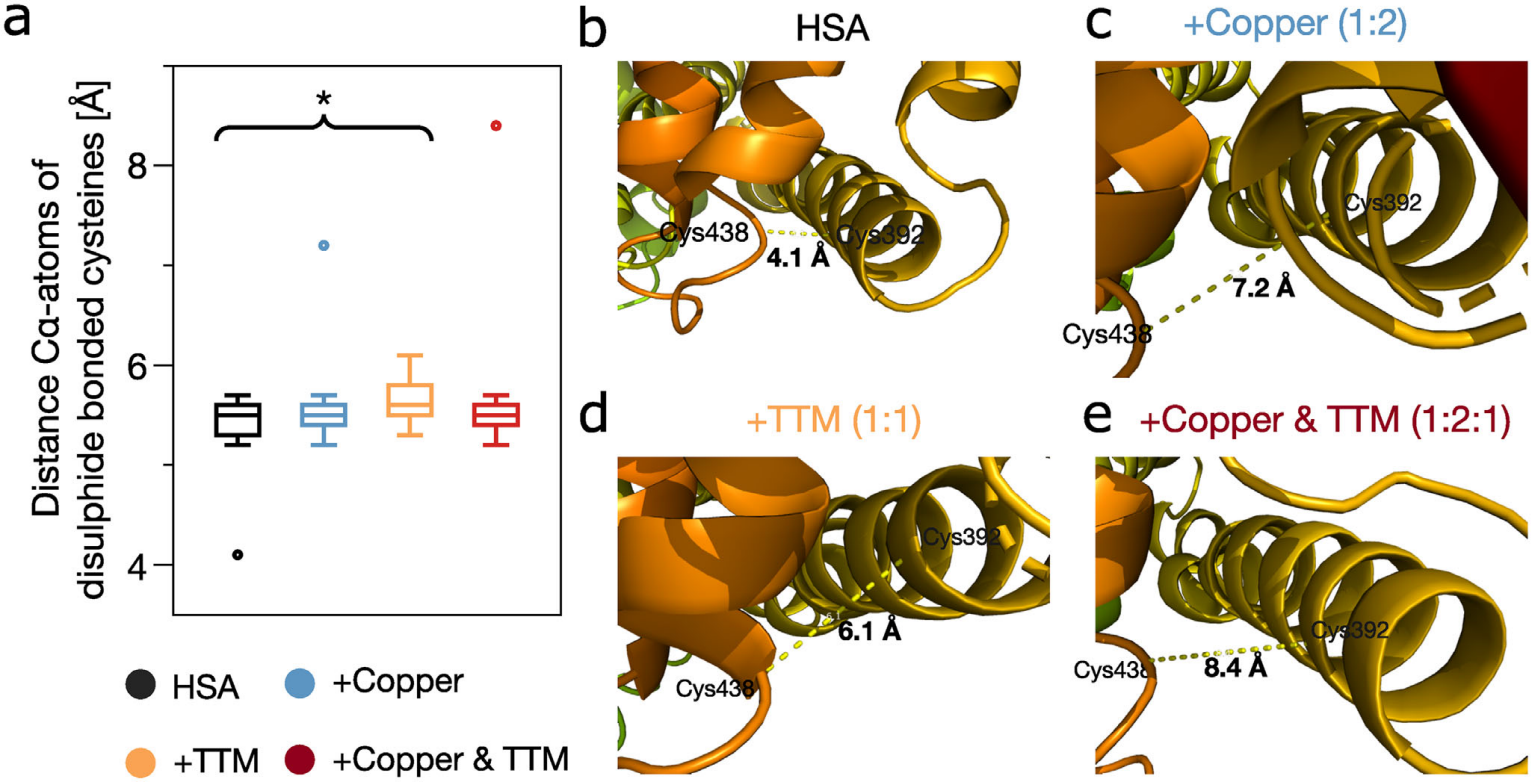
SAXS modelling reveals domain III disulphide Cys392–Cys438 is structurally perturbed during copper redox modulation (a) Quantification of average Cα–Cα distances across all 17 disulphide bonds in HSA based on SAXS‐refined models under different conditions: apo HSA (black), with ${\mathrm{CuCl}}_{2}$ (blue), with TTM (orange) and with both ${\mathrm{CuCl}}_{2}$ and TTM (red). While most disulphide bond distances remain stable, a significant expansion is observed in the TTM‐treated sample (*p*=0.0026). The primary outlier was the Cys392–Cys438 bond, indicating selective disulphide perturbation. (b–e) Structural visualisation of the Cys392–Cys438 disulphide bond from SAXS‐refined HSA models. The Cα–Cα distance increases progressively from the native state (b, ∼4.1 Å) to Copper (c, ∼7.2 Å), TTM (d, ∼6.1 Å) and Copper+TTM (e, ∼8.4 Å), exceeding typical disulphide geometries and supporting redox‐active function.

We next analysed disulphide bond geometry using SAXS‐refined models to identify potential redox‐active sites. Among the 17 disulphide bonds in HSA, most of which are buried in stable α‐helical regions, Cys392 and Cys438 stood out in SAXS‐refined models (Figures 3 and S9). The Cα–Cα distance between these residues increased from 4.1 Å  in the apo‐state to 7.2 Å  with copper and 8.4 Å  with both copper and TTM. These values exceed typical disulphide bond length [54], suggesting destabilisation or partial cleavage.

This bond is located in domain III, which undergoes the most prominent conformational rearrangements in SAXS. No comparable distance changes were observed in other disulphide bonds. This site is also structurally positioned near His440 and Arg445, potentially facilitating interaction with copper. This structural evidence, combined with CD and SAXS data showing compaction and disulphide rearrangement in domain III, suggests that Cys392–Cys438 is most likely involved in Cu(II) reduction. The domain's flexibility and proximity to known fatty acids and metal‐binding sites make it a plausible reduction site, likely regulated via allosteric changes triggered by copper binding. We propose that Cu(II) gains access to this site through a conformational change, promoting disulphide bond cleavage. Electron transfer is likely facilitated by thiol‐disulphide exchange [55], leading to Cu(I) formation. TTM binding may hinder the process by forming a stable complex and triggering an allosteric rearrangement of domain III. Alternatively, steric shielding around the Cys392–Cys438 region could account for the reduced reactivity, or Cu–TTM complex formation might reduce the pool of Cu(II) available for redox exchange.

### 2.4 Copper Reduction Depends on Disulphide Accessibility Across Globular Proteins

**FIGURE 4:**
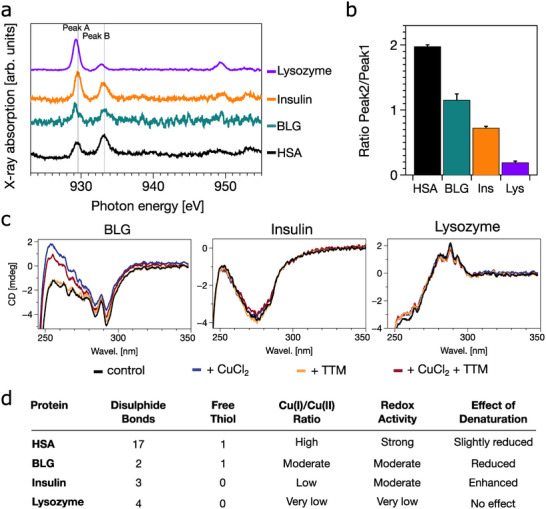
Copper redox activity across globular proteins correlates with disulphide accessibility and structural flexibility. (a) Soft x‐ray absorption spectra at the Cu $L_{2,3}$‐edges for HSA (black), β‐lactoglobulin (BLG, green), insulin (orange) and lysozyme (purple), each incubated with ${\mathrm{CuCl}}_{2}$ (molar ratio 1:2) at pH 7.4. Cu(I)‐associated features are most pronounced in HSA and weakest in lysozyme. (b) Quantified Cu(I)/Cu(II) ratios derived from integrated peak areas of (a). HSA exhibits the highest Cu(I) proportion; lysozyme the lowest. (c) Near‐UV CD spectra comparing protein structural changes upon copper and TTM addition. Disulphide sensitive signals (∼320 nm) shifts significantly in HSA (Figure 2) and slightly in BLG, and not in insulin and lysozyme. (d) Summary table comparing disulphide architecture, free thiol presence, redox activity and denaturation sensitivity for each protein. HSA combined high redox activity with flexible disulphide topology; lysozyme, with tightly packed disulphides an no free thiols, shows no redox response.

To evaluate whether copper reduction is unique to HSA or a more general property of disulphide‐containing globular proteins, we examined three other globular proteins; β‐lactoglobulin (BLG), insulin, and lysozyme using XAS and CD spectroscopy (Figures 4a and S10). These proteins vary in disulphide content, flexibility and copper‐binding affinity. BLG contains two disulphide bonds and one free thiol [56], insulin contains three disulphide bonds and no free thiols [57], and lysozyme contains four disulphide bonds [58] (Figures 4d and S11).

Soft XAS spectra showed that BLG and insulin exhibit weak Cu(I) signals, while lysozyme showed almost none. CD spectra indicated minor structural rearrangements in BLG and insulin upon Cu or TTM binding, but lysozyme remains unchanged. However, none showed an alteration of the disulphide bonds. In general, HSA showed the strongest binding affinity to copper, while lysozyme exhibited the lowest [35, 59, 60, 61, 62].

Notably, heat denaturation abolished Cu(II) reduction activity, indicating that native fold is essential. Heat‐induced denaturation leads to the loss of native structure and the formation of large amorphous aggregates [63]. As a result, all proteins display reduced Cu‐binding capacity. This may be due either to disruption of key disulphide bond positioning or to loss of allosteric communication required for redox activation. Among the proteins tested, only HSA exhibited robust redox modulation with associated conformational rearrangements. Insulin's reduced Cu(II) affinity upon dimerisation has been previously documented [61]. This highlights the importance of both disulphide topology and structural flexibility in enabling protein‐driven copper redox activity.

### 2.5 Disulphide Cleavage in SOD1 Enhances Copper–Cysteine Interactions and Facilitates Redox Modulation

**FIGURE 5:**
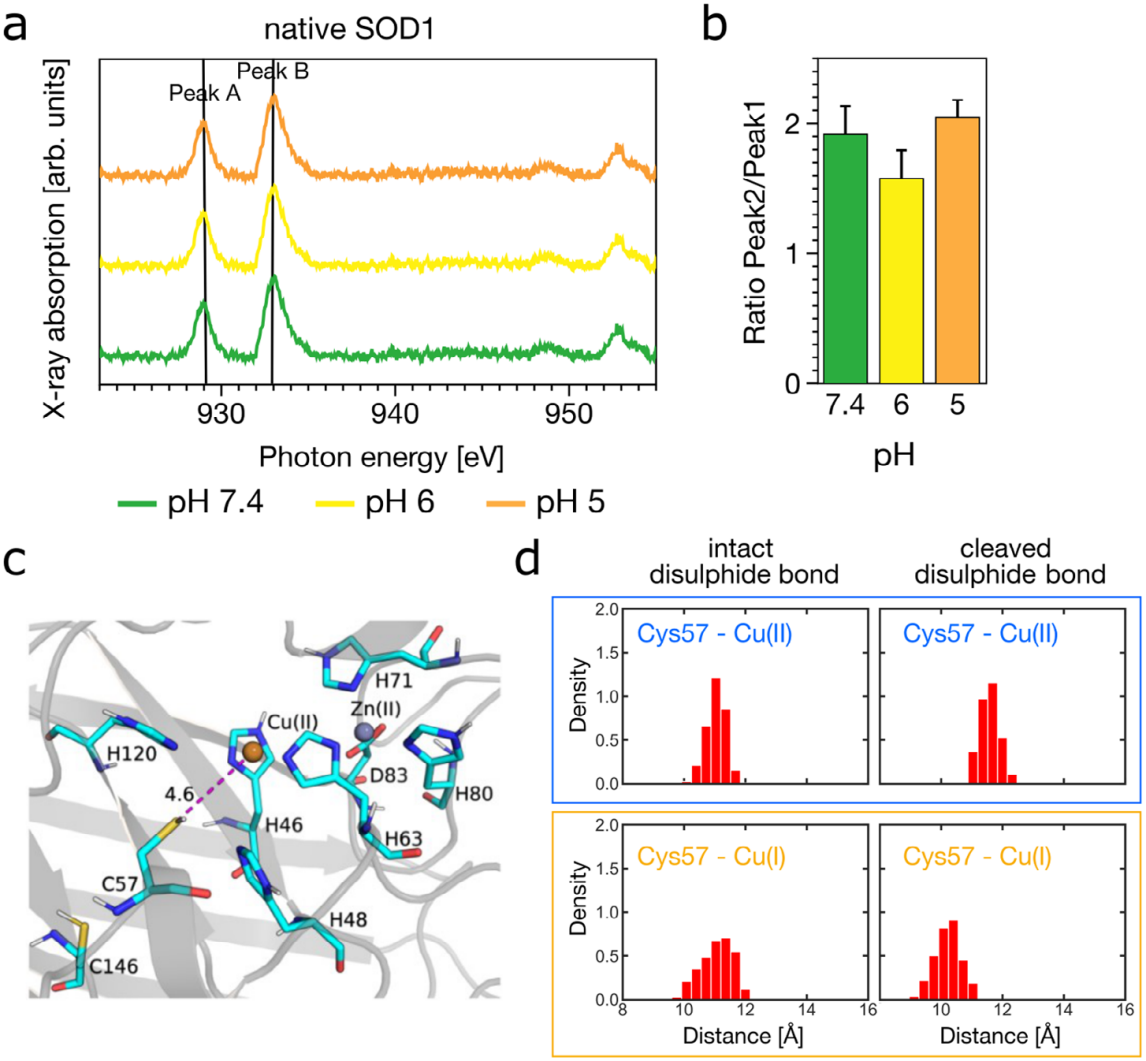
Disulphide bond cleavage in SOD1 enhances Cu–thiol interaction and facilitates Cu(II) reduction. (a) Soft XAS spectra of native SOD1 at pH 7.4 (green), 6.0 (yellow) and 5.0 (orange), showing consistent Cu(I)/Cu(II) ratio, indicating that histidine protonation alone does not modulate copper redox state. (b) Ratio of Cu(I) to Cu(II) peak areas extracted from (a), confirming stable redox state across pH conditions. (c) QM/MM MD simulations show that copper approaches Cys57 closely, when the Cys57–Cys146 disulphide bond is cleaved. (d) Histograms showing Cu–Cys57 distances from QM/MM MD simulations. Disulphide cleavage shortens the Cu–S distances, especially in the Cu(I) state, presumably enhancing Cu–thiolate coordination.

To validate the role of disulphide bonds in copper redox modulation, we studied superoxide dismutase 1 (SOD1) as a comparative model. SOD1 requires a bound copper ion, which can cycle between two oxidation states, enabling its function [59, 64]. Soft XAS measurements across a mildly acidic pH range (pH 5.0–pH 7.4) showed stable Cu(I)/Cu(II) ratios, indicating that histidine protonation alone does not significantly affect copper oxidation state (Figure 5). This aligns with our observations in HSA, where redox activity was linked instead to disulphide bond dynamics.

Classical MD simulations showed that in native (oxidised) SOD1, Cu remains distant from redox‐active cysteines (Cys57/Cys146) (Figure S12). Upon disulphide bond cleavage, the observed migration of Cu(II) closer to Cys57 suggests a potential increase in Cu‐S interaction. However, the resulting 4–5 Å  separation is inconsistent with classical Cu(II)–thiolate coordination. This proximity may reflect a transient, pre‐coordination state; sampling further reduction in distance required for stable coordination lies beyond our present computational reach.

These findings support a model in which disulphide bond cleavage enables Cu‐thiolate interaction and electron transfer. The same mechanism likely operates in HSA at Cys392–Cys438, suggesting that disulphide‐gated copper redox activity is a generalizable feature in globular proteins.

## 3 Discussion

Transition metals are essential to many biological processes, from energy production to oxidative stress response and intracellular signalling. Copper, in particular, has critical roles in neurotransmitter synthesis and oxidative stress regulation, but disruptions in copper homeostasis can lead to neurotoxicity and disease. Traditionally, protein‐mediated Cu(II) reduction has been associated with pathological or unfolded proteins such as amyloid proteins, mediated primarily by His, Trp and Cys residues [19, 65, 66]. However, our findings demonstrate that well‐folded globular proteins like HSA also possess intrinsic Cu(II)‐reducing capability, independent of classical reducing agents.

HSA, the most abundant protein in plasma, is a primary copper transporter. While only a small fraction of circulating HSA typically carries copper [67], this increases in disorders such as Wilson disease [68], where copper‐bound HSA contributed to endothelial damage and blood–brain barrier disruption [69]. Previous studies have shown that HSA can bind both Cu(II) and Cu(I) [47], the latter usually formed via chemical reduction leading to a digonal conformation [28, 47]. Here, we show that Cu(I) also forms in HSA without external reductants, and exhibits distinct coordination from ascorbate‐reduced Cu(I), suggesting distinct intrinsic reduction site.

As a first thought, the involvement of the conserved residue Cys34, the only free thiol in HSA, which constitutes the largest pool of reactive thiols in plasma, appears to be the most obvious. Its thiol group, typically reduced [70], is essential to protect against oxidative stress, which can lead to a significant increase in oxidised Cys34 (from 35% to 70% [71]). Cysteines are efficient Cu(II) reductants [8, 9] and important low molecular mass chelator in blood plasma [10]. Apart from methionine residues, cysteines are the most common amino acid in proteins, with abundances in mammals of approximately 2.03%–2.45% [72], affecting the protein structure, stability and function [73, 74, 75]. Although Cys34 acts as a physiological anti‐oxidant and therefore represents a plausible candidate for intrinsic Cu(II) reduction, our SAXS and structural data show no detectable conformational changes in domain I, where both Cys34 and the ATCUN motif reside. This strongly suggests that the structural environment around Cys34 remains unchanged during the reduction process. Moreover, Cu(I) generated by Cys34 would be expected to adopt linear bis‐Cys coordination geometries [47] as observed for ascorbate‐reduced Cu(I), whereas the Cu(I) species formed intrinsically by HSA displays a four‐coordinate geometry, consistent with involvement of residues in domain III, such as Cys392, Cys438 and His440, Arg445 and Lys389. Taken together, these observations indicate that while Cys34 may contribute to the overall redox buffering capacity of HSA, the intrinsic Cu(II) to Cu(I) conversion observed here proceeds via a spatially and functionally distinct site, located in domain III and structurally independent both of Cys34 and the ATCUN region.

Using SAXS‐guided modelling and chemical probing, we identified the disulphide bond Cys392‐Cys438 in domain III as a likely redox‐active site. The substantial lengthening of this disulphide bond upon Cu and Cu/TTM binding reflects a local metal coordination‐induced structural rearrangement that modulates the redox micro‐environment, reducing cysteine accessibility as evidenced by decreased disulphide occupancy with TTM (Figure 2g). Reversible thiol modifications, including S‐cysteinylation of Cys392 and Cys438, have been observed in HSA from hyperlipidaemia patients [76] and hyperactivity could be observed [77], supporting their potential involvement in redox activity. QM/MM simulations support the hypothesis that disulphide bond cleavage might facilitate Cu–thiolate interaction and electron transfer, leading to Cu(II) reduction. These data suggest a redox switching mechanism gated by disulphide accessibility.

TTM, a copper chelator and therapeutic agent, interferes with this mechanism. While it stabilises HSA thermally and induces structural compaction, it also alters redox activity and structural dynamics in domain III. The conformational closure observed between domain I and III might disrupt access to the Cys392–Cys438 region, thereby inhibiting copper reduction. These findings imply that TTM may modulate not only Cu bioavailability but also protein structure and function, potentially affecting ligand binding at known pharmacological sites, that is, by closing the gap between the subdomains IA–IB–IIA and IIB–IIIA–IIIB.

As shown in Figures 4d and S11, comparison with other globular proteins reinforces that the mechanism observed for HSA is not a generic property of folded proteins but instead reflects HSA‐specific disulphide topology and structural dynamics. β‐lactoglobulin (BLG) and insulin exhibited only modest redox activity, whereas lysozyme shows none at all, despite all three proteins containing multiple disulphide bonds. This variability suggests that not all disulphides are positioned or strained in a way that allows electron transfer to bound Cu(II). In BLG, most disulphide bonds are deeply buried, restricting solvent accessibility and geometric distortion. Disulphide bonds in insulin, while partly exposed, are conformationally rigid and primarily structural, which limits their ability to undergo redox‐driven reshaping. All disulphide bridges in lysozyme are integrated into its rigid fold, leading them to not be redox‐active under physiological conditions. Our denaturation experiments further demonstrate that both fold architecture and disulphide geometry are critical determinants of Cu(II) reduction. By contrast, HSA‐mediated Cu(II) reduction remains relatively persistent even after thermal denaturation, which likely reflects the exceptional stability and solvent exposure of particular HSA disulphide bonds that retain redox functionality even when tertiary structure is compromised [34]. Together, these observations indicate that HSA possesses a uniquely permissive disulphide configuration, enabling a functional disulphide‐mediated redox cycle that is absent or greatly diminished in other globular proteins.

We further validated the proposed disulphide‐mediated Cu(II) reduction mechanism in SOD1, a classical metalloprotein containing a structurally integral Cu site and an internal disulphide bond (Cys57–Cys146). XAS data showed that the Cu redox state remains stable over a broad pH range, ruling out histidine protonation as a primary driver. QM/MM simulations indicated that disulphide cleavage enhances proximity between Cu and Cys57 in picosecond time frame. These results support a model in which disulphide dynamics regulate copper redox state through changes in site accessibility and coordination geometry.

Mechanistically, this is conceptually similar to the domain III disulphide‐mediated reduction observed in HSA. In both cases, electron transfer from disulphide contributed to Cu(I) formation. Key differences, however, reflect the distinct functional contexts. HSA is a transport protein in which domain III flexibility is required to bring Cu(II) into proximity with the reactive disulphide, whereas SOD1 is an enzymatic protein with a rigid fold, the Cys57–Cys146 disulphide bond is structurally integral and positioned close to the active‐site Cu, allowing efficient electron transfer without large‐scale conformational rearrangements. Notably, known SOD1 variants and post‐translational modifications alter the stability or local environment of this disulphide bond [78, 79, 80, 81, 82]. These examples highlight the mechanistic importance of the Cys57–Cys146 disulphide bond for Cu(I) formation, without extrapolating to disease outcomes such as ALS.

**FIGURE 6:**
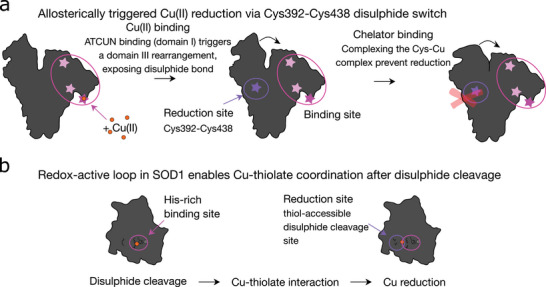
Mechanistic model of copper reduction in globular proteins via disulphide‐gated redox switching.(a) In HSA, Cu(II) binds to the high‐affinity ATCUN motif (domain I), triggering an allosteric structural rearrangement in domain IIIA/B. Future deletion studies of the ATCUN motif would substantiate this mechanism, such experiments are not feasible within the current work. This exposes the disulphide bond Cys392–Cys438, enabling thiol coordination and electron transfer. The Cu(II) is subsequently reduced to Cu(I) in a four‐coordinate geometry. Chelation by tetrathiomolybdate (TTM) may prevent this reduction by forming a stable complex that induces an allosteric rearrangement of domain III, sterically shielding the Cys392–Cys438 region, or lowering the pool of Cu(II) available for redox exchange. (b) In SOD1, Cu(II) is initially bound at the histidine‐rich active site. Cleavage of the intramolecular disulphide bond (Cys57–Cys146) enables Cu to shift toward Cys57, enhancing thiolate interaction and facilitating Cu(I) formation. This supports a dynamic redox loop that is modulated by structural integrity and disulphide status.

Taken together, we propose a general mechanism in which high‐affinity Cu(II) binding initiates structural changes that expose a disulphide‐containing secondary site. Cleavage of this bond permits Cu–thiolate coordination and facilitates electron transfer. This two‐site model, exemplified by both HSA and SOD1 (see Figure 6), suggests that redox regulation by disulphide bonds may be a broader feature of globular proteins than previously appreciated.

Our findings supported by data, demonstrate that HSA mediated intrinsic Cu(II) reduction through a structurally defined disulphide bond in domain III. SOD1 shows analogous disulphide‐mediated Cu(I) formation, with QM/MM and XAS results confirming the mechanistic role of the Cys57–Cys146 disulphide bond. Structural specificity and disulphide geometry, rather than simply the presence of cysteins, determine redox activity, as demonstrated with BLG, insulin and lysozyme. TTM binding modulates HSA structure and inhibits Cu(II) reduction, showing that protein conformation can regulate copper redox chemistry.

Beyond these conclusions, it is tempting to speculate on physiological or pathological implications. Transition metals such as copper play critical roles in energy metabolism, neurotransmitter synthesis, and oxidative stress regulation, and dysregulation of Cu homeostasis can contribute to toxicity and disease [1, 5]. It is conceivable that disulphide‐mediated redox mechanisms in HSA and potentially other plasma proteins could influence copper trafficking, allosteric regulation or redox balance in vivo. Such mechanisms might also intersect with disease states characterised by copper imbalance, including WD, MD or neurodegenerative conditions. We emphasise that these points are hypothesis‐driven and are not directly tested by our experiments but suggest directions for future studies.

In summary, our work expands our understanding of protein‐based redox chemistry and copper biology. By uncovering a structural mechanism for Cu(II) reduction in folded proteins, this work opens new directions for studying copper trafficking, allosteric control, and redox regulation in physiological and pathological settings. Further research may explore whether other disulphide‐containing plasma proteins employ similar redox switches, and how these mechanisms interface with disease states involving copper imbalance.

## 4 Conclusion

Our results uncover a novel copper redox mechanism in globular proteins driven by structural rearrangements and disulphide bond dynamics. In HSA, high‐affinity Cu(II) binding initiates allosteric changes that expose a reactive disulphide site, enabling electron transfer and Cu(I) formation. This mechanism is impaired by chelators like TTM and absent in proteins with inaccessible disulphides or rigid structures. Validation in SOD1 supports the generality of disulphide‐gated copper reduction. These findings broaden the known scope of redox activity in plasma proteins and suggest new avenues for exploring copper regulation in health and disease.

## Author Contributions

R. S. H. and J. L. conceived the experiments. R. S. H., C. L., X. W., H. P., X. S. and P. P. conducted the experiments. M. K., Q. L and J. L. performed and analysed computer simulations. R. S. H. and C. L. analysed the results. J.L. supervised the project. R. S. H. and J. L. wrote the manuscript. All authors reviewed the manuscript.

## Conflicts of Interest

The authors declare no conflicts of interest.

## Supporting information


**Supporting File 1**: anie71493‐sup‐0001‐SuppMat.pdf.

## Data Availability

The data that support the findings of this study are available from the corresponding author upon reasonable request.
