# Supplementary material for: Protein‐Driven Copper Redox Regulation: Uncovering the Role of Disulphide Bonds and Allosteric Modulation
Source: Angew Chem Int Ed Engl. 2026 Feb 27;65(15):e19673. doi: 10.1002/anie.202519673 (PMC13053925; doi:10.1002/anie.202519673)
Supplement: Supplementary file 1 — Supporting File 1: anie71493‐sup‐0001‐SuppMat.pdf. [file ANIE-65-e19673-s001.pdf]

Protein-Driven Copper Redox Regulation: Uncovering the Role of Disulphide Bonds and Allosteric Modulation

Rebecca Sternke-Hoffmann[1], Chang Liu[1], Xue Wang[1], Hegne Pupart[2], Xun Sun[1], Jan Gui-Hyon Dreiser[3], Peep Palumaa[2], Qinghua Liao[4], Matthias Krack[5], and Jinghui Luo[1,*]

[1]PSI Center for Life Sciences, 5232 Villigen PSI, Switzerland
[2]Department of Chemistry and Biotechnology, Tallinn University of Technology, 12618 Tallinn, Estonia
[3]PSI Center for Photon Science, 5232 Villigen PSI, Switzerland
[4]Departament de Química Inorgànica i Orgànica (Secció de Química, (Orgànica)Institut de Química Teòrica i Computacional (IQTCUB), Universitat de Barcelona, 08028 Barcelona, Spain
[5]PSI Center for Scientific Computing, Theory and Data, 5232 Villigen PSI, Switzerland
*jinghui.luo@psi.ch

Additional information

This Supporting Information provides the experimental section, complementary data and computational analyses that expand on the experimental findings presented in the main manuscript. It includes additional X-ray absorption spectra (Fig. S2, S1) confirming copper oxidation states in HSA and peptide complexes, and extended SAXS and biophysical characterisations (Fig. S3, S4, S5, S6, S7, S8, S9) detailing structural changes, induced by copper and TTM. Fig. S10 extends the XAS analysis to other globular proteins under denaturing conditions, revealing how structural destabilisation influences copper redox behaviour. Structural representations of the globular proteins including their disulphide bonds are shown in Fig. S11. Figure S12 offers computational insights, including MD simulations and QM/MM, supporting the proposed mechanism of disulphide-gated copper reduction in globular proteins. Together, these data reinforce the structural and redox dynamics underlying copper-protein interactions and provide a broader framework for interpreting redox behaviour across different protein classes.

Methods

Sample preparation

Proteins used in this study, e.g. HSA, BLG, insulin and lysozyme were purchased from Sigma-Aldrich A3782, L2506, I2643 and L1667, respectively. The proteins were dissolved in 20 mM HEPES buffer, pH 7.4 at a concentration of 1.5 mM and subsequently purified using a Superdex 75 increase on a NGM system (Biorad). The same buffer was used as a running buffer. The high concentrated samples were aliquoted, frozen with liquid nitrogen, and stored at -20°C until further use. The SOD1 protein was expressed and purified by established protocols[1]. Briefly, SOD1 was expressed in BL21(DE3)pLysS competent cells and after harvesting the cells were lysed by sonication (3x 1 min on ice, 1 s on, 0.5 s off). The cleared lysate was incubated at 65°C for 30 min in a water bath and centrifuged. The SOD1 was precipitated with ammonium sulphate in three steps (50%, 60% and 90%) and subsequently purified using a HiLoad Superdex75 16/60 column and Hitrap Q FF column. DAH-NH2, DTHFPI-NH2, and MEHFPGP-NH2 were synthesized at the Institute of Technology of the University of Tartu, Estonia. The peptides were synthesized on an automated peptide synthesizer (Biotage Initiator+ Alstra, Sweden) using a fluorenylmethyloxycarbonyl (Fmoc) solid-phase peptide synthesis strategy and purified by reverse-phase liquid chromatography on a C4 column (Phenomenex Jupiter C4, 5 µm, 300 A, 250 x 10 mm) using a gradient of acetonitrile/water containing 0.1% TFA. The molecular weight of the peptides was determined by a MALDI-TOF mass spectrometer (Bruker Microflex LT/SH).

Soft X-ray absorption spectroscopy

The samples for soft XAS measurements were diluted to a final concentration of 100 µM, supplemented with 200 µM $CuCl_2$ and in the samples to investigate the chelating mechanism of TTM additional 100 µM TTM for a final molecular ratio of 1:2:1 (protein:$CuCl_2$:TTM). The samples were incubated for 1 to 2 h at room temperature and deposited on the surface of a silicon

dioxide wafer. The deposited samples were immediately dried with nitrogen gas to form a thin film. Protein samples denoted as heat denatured, were incubated 80°C for 30 min before $CuCl_2$-addition. Additional metal ions were not added to SOD1 prior measurement. SOD1 was purified according to established protocols[1]. After purification, SOD1 was diluted to 100 µM and immediately deposited on a silicon dioxide wafer or incubated with lipids DOPC:DOPG (4:1) for 1 h at 37°C (lipid/SOD1 ratio: 15/1) before drying. The lipid vesicles were prepared by dissolving 11.25 mg/ml 1,2-dioleoyl-sn-glycero-3-phosphocholine (DOPC), 1,2-dioleoyl-sn-glycero-3-phospho-(1'-rac-glycerol) (DOPG) (4:1) in 100 mM NaCl solution. The lipids were dried with nitrogen gas on a glass bottle surface and then with a desiccator for 2-3 hours. Afterwards 100 mM NaCl solution was added, vortexed for 5 min and then sonicated for 15 min to generate lipid vesicles with smaller and homogeneous size.

The XAS spectra were recorded at the X-Treme beamline[2] at the Swiss Light Source, Paul Scherrer Institut, Switzerland. To protect the samples from beam damage, an attenuated photon flux and a defocused X-ray spot (0.5 mm^2) on the sample were chosen. Specifically, the impinging X-ray photon flux per area was 0.06 photons/sec/nm^2 at the Cu $L_{2,3}$-edges. The spectra were recorded over a timescale of several tens of minutes and when changes were observed, the measurement time was reduced and fresh sample spots were recorded for repeats. The spectra were recorded at room temperature in normal incidence of the X-ray beam in the total electron yield mode (TEY) using on-the-fly scanning. Spectra were normalized for the sum of the peak areas to yield a constant of 1.0 after subtracting the baseline. Reference spectra at the Cu $L_{2,3}$-edges were obtained on a drop-cast film of Cu(II)-phthalocyanine on silicon dioxide wafer.

Hard X-ray absorption spectroscopy

Hard X-ray absorption spectroscopy in fluorescence mode was conducted at the SuperXAS beamline at the Swiss Light Source, Paul Scherrer Institut, Switzerland. The samples were prepared and analysed in aqueous solution with a protein ratio of 1:2 to $CuCl_2$. The final $CuCl_2$ concentration was 1 to 1.5 mM. After an incubation of 1 to 2 h, the samples were loaded into the sample holder and flash frozen with liquid nitrogen. The XANES of the Cu K-edge was recorded using a cryoholder. 5 spectra per sample were recorded and analysed using the XAS data processing tool Athena.

MD simulation

Two Cys residues with copper ions in the SOD1 structural model

In order to explore the dynamics between the two CYS residues with copper ion, four systems were set up for the SOD1 as structural model. One is for SOD1/Cu(II) with and without a disulphide bond between C57 and C146 (referred as SOD1/Cu(II)/SS and SOD1/Cu(II)/SHSH), the other one is SOD1/Cu(I) with and without a disulphide bond between C57 and C146 (referred as SOD1/Cu(I)/SS and SOD1/Cu(I)SHSH). The crystal structure of SOD1 (PDB ID: 1HL5[3], chains A and H) was used as the starting structure for all simulations of the four systems reported in this work. The simulations were performed at pH 7.0. The protonation states of His were chosen based on hydrogen bond network and ions coordination manually examined, H46, H71 and H80 are protonated at Nε, H48 and H120 are protonated at Nδ, while H63 is double deprotonated as it is coordinated with both Zn^{2+} and Cu^{2+}. All Asp and Glu residues were negatively charged while all Arg and Lys residues were positively charged. The protein was immersed in a water box with a 10 Å buffer of TIP3P water molecules[4], and Na^+ and Cl^- were added to neutralize the system and maintaining 0.15 mol/L NaCl mimicking the physiological condition. The protein was described using the Amber ff14SB force field, while the Lennard-Jones 12-6-4 potential of Zn^{2+} and Cu^{2+}[5] against TIP3P water model were used [ref: Li]. The LEaP module of AmberTools 24[6] was used to generate the topology and coordinate files for the classical MD simulations, which were carried out using the CUDA version of the PMEMD module of the AMBER 24 simulation package[7]. The solvated system was first subjected to 5000 steps steepest descent minimization, followed by 5000 steps conjugate gradient minimization with positional restraints on all heavy atoms of the solute, using a 50 kcal $^{-1}$ Å^{-2} harmonic potential. The minimized system was then heated up to 300 K using the Berendsen thermostat, with a time constant of 1 ps for the coupling, and 50 kcal $^{-1}$ Å^{-2} positional restraints applied over three 500 ps steps of heating process. The positional restraints were then gradually decreased to 5 kcal mol^{-1} Å^{-2} over five 500 ps steps of NPT equilibration, using the Berendsen thermostat and barostat to keep the system at 300 K and 1 atm. For the production run, each system was subjected to 100 ns of sampling in an NPT ensemble at constant temperature (300 K) and constant pressure (1 atm), controlled by the Langevin thermostat, with a collision frequency of 2.0 ps^{-1}, and the Monte Carlo barostat with a coupling constant of 1.0 ps. The SHAKE algorithm[8] was applied to constrain all bonds involving hydrogen atoms. A cut-off of 9.0 Å was applied to all non-bonded interactions, with the long-range electrostatic interactions being treated with the particle mesh Ewald (PME) approach[9]. A time step of 2 fs was used for all the classical simulations, and coordinates were saved from the simulation every 5 ps. Three independent runs were performed.

QM/MM MD simulation

To further access the interplay between the Cu (II/I) and the two CYS residues, quantum mechanics/molecular mechanics (QM/MM)[10] MD simulations were performed for both of the four systems. ACPYPE[11] was used to convert the Amber topology and coordinates into the Gromacs format forms. The last snapshot from the classical MD trajectory at simulation time of 100 ns

was then used for the subsequent QM/MM MD simulations, which combines Born-Oppenheimer MD simulation, based on density functional theory (DFT), with force-field MD methodology. For SOD1/SS, the QM region consists of the sidechains of H46, H48, H63, H71, H80, D83, H120 and copper as well as zinc ions, resulting in a QM region of 80 atoms (including 7 capping hydrogens), as shown in Fig. S13a. Apart from QM atoms in SOD1/SS, the sidechains of C57 and C146 are added to the QM region for SOD1/SHSH, leading to a total of 92 atoms in the QM region including 9 capping hydrogens (Fig. S12). The QM atoms were centred in a QM box of 18×18×18 $Å^3$ for SOD1/SS and 24×24×16 $Å^3$ for SOD1/SHSH. All QM/MM MD simulations were performed using Gromacs 2022.5[12] interfaced with CP2K 2024.1[13,14], combining the QM program QUICKSTEP[15] of CP2K and the MD engine of Gromacs. In this code, a real space multigrid technique is used to compute the electrostatic coupling between the QM and MM region. The QM region was treated at the DFT (BLYP) level, employing the dual basis set of Gaussian and plane-waves (GPW) formalism, whereas the remaining part of the system was modelled at the classical level using the same parameters as in the classical MD simulations. The Gaussian triple-ζ valence polarized (TZV2P) basis set was used to expand the wave function, while the auxiliary plane-wave basis set with a density cut-off of 400 Ry and GTH pseudopotentials[16,17] was utilized to converge the electron density. All QM/MM MD simulations were performed under the NVT ensemble at a constant temperature of 300 K using velocity rescaling thermostat with a coupling constant of 50 fs^{-1} and an integration time step of 0.5 fs. First, the system was minimized using the steepest descent method, then it is further equilibrated without any constraint for 5.0 ps. Afterwards, the production runs were performed 56 ps for SOD1/SS and 25 ps for SOD1/SHSH.

Radius measurement

The radius was determined using the Panta Prometheus (Nanotemper). The samples were prepared to a final concentration 100 µM protein supplemented with different concentration of $CuCl_2$, TTM or both using 20 mM HEPES, pH 7.4. The samples were loaded into Prometheus Series capillaries (PR-C002) and the DLS signal was measured.

Thermodynamic stability

The thermodynamic stability was determined using the scattering as a readout from the Panta Prometheus (Nanotemper). The samples prepared in the same way as for the radii measurements were loaded into Prometheus Series capillaries (PR-C002), closed and heated up from 25°C to 90°C with a scanning rate of 1°C/min.

CD spectroscopy

CD spectra were recorded at 20°C using a Chirascan plus with a temperature control Quantum Northwest. Final protein concentration was 100 µM or 200 µM. Differences in secondary structure were first examined, for HSA as an example using PB instead of HEPES, since no influence of $CuCl_2$ or TTM on the secondary structure could be observed, the changes in the near-UV were investigated, recording spectra from 250 nm to 350 nm.

Small angle X-ray scattering

SAXS spectra were collected on the EMBL beamline P12[18] at Petra III at DESY (Deutsches Elektronen-Synchrotron), Hamburg, Germany. The samples were shipped in liquid nitrogen. Buffer subtraction and data processing were performed using the ATSAS software package, including PRIMUS, to determine the radius of gyration (R_g). Different pdb structures were fitted to the SAXS curves using CRYSOL and flexible refinement using SREFLEX was performed mainly using the models 3b9l (pdb), 4LA0 (pdb), SASDAA6 (SASBDB) and an alphafold model. Since the models lead to rather similar χ^2 values, the presented SREFLEX refinement was performed using model 4LA0 and an alphafold model AF-P02768-F1-v4. 4LA0[19] is a X-ray structure of HSA complexed with bicalutamide and is one of the few structures containing the AH residues of the ATCUN motif. For the model refinement, the first 30 points were skipped. A consensus structural model was calculated from the best-fitting models based on their x^2 values. The models were aligned using SUPCOMB and an average model was calculated using PyMOL. The average models were fitted against to the SAXS curves using CRYSOL.

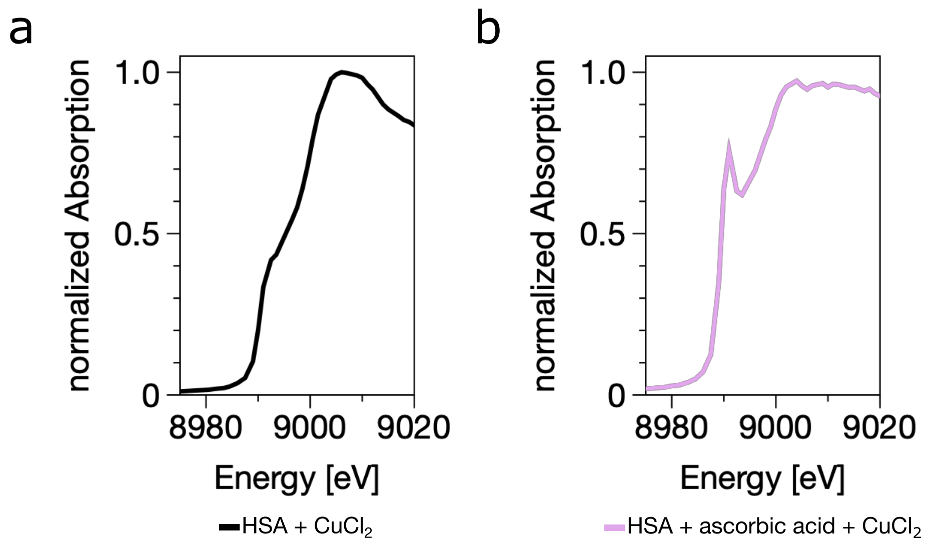

Figure S1. Hard x-ray spectroscopy confirms Cu(I) species within HSA (a). However, Cu(I) formed via ascorbate reduction (b) exhibits a distinct coordination geometry, indicating different copper-binding environments.

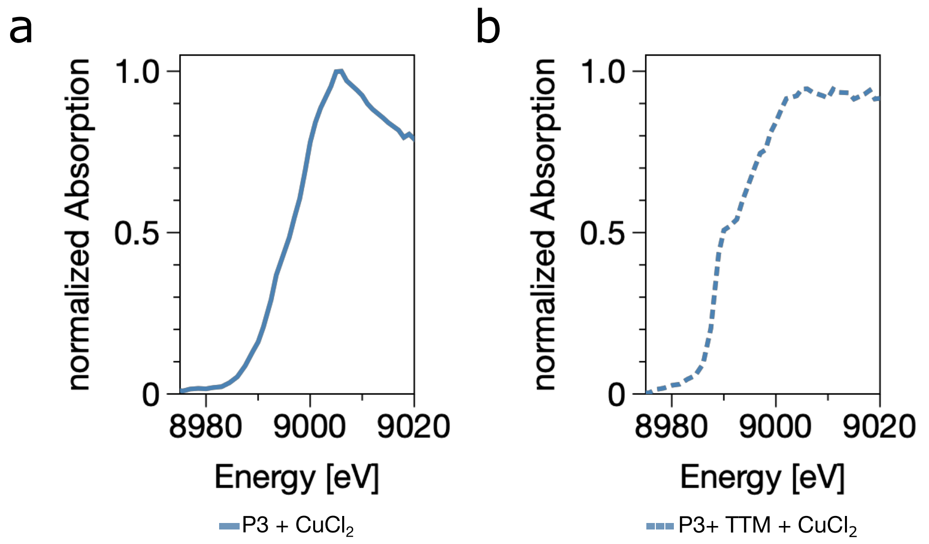

Figure S2. Hard x-ray absorption spectroscopy at the Cu K-edge reveals Cu(II) in peptide 3 (MEHFPGP) (a), while the addition of TTM promotes Cu(I) formation (b).

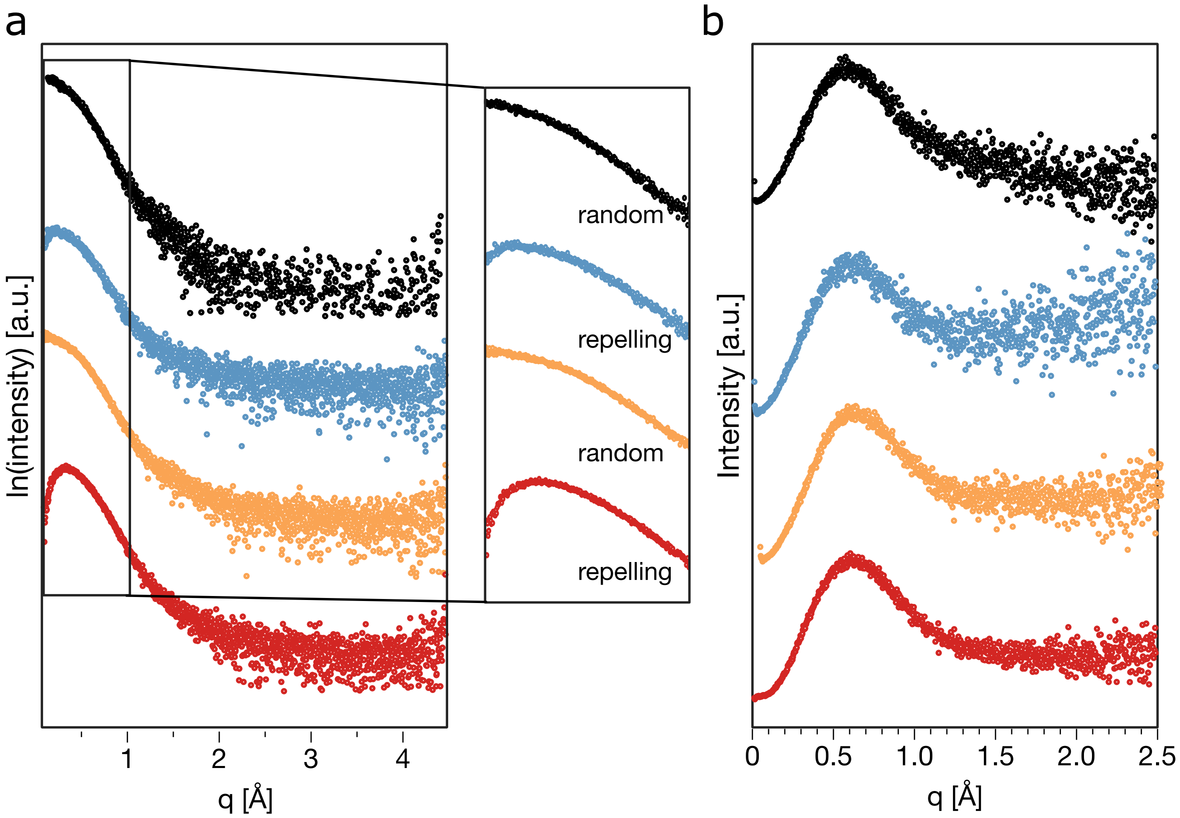

Figure S3. (A) Static Small-angle X-ray scattering (SAXS) profiles of HSA (black), with $CuCl_2$ (blue), with TTM (orange) and both (red). Copper induces a repulsive particle distribution; TTM alone does not. (b) Kratky plots indicate that all samples remain folded, though copper increases flexibility slightly.

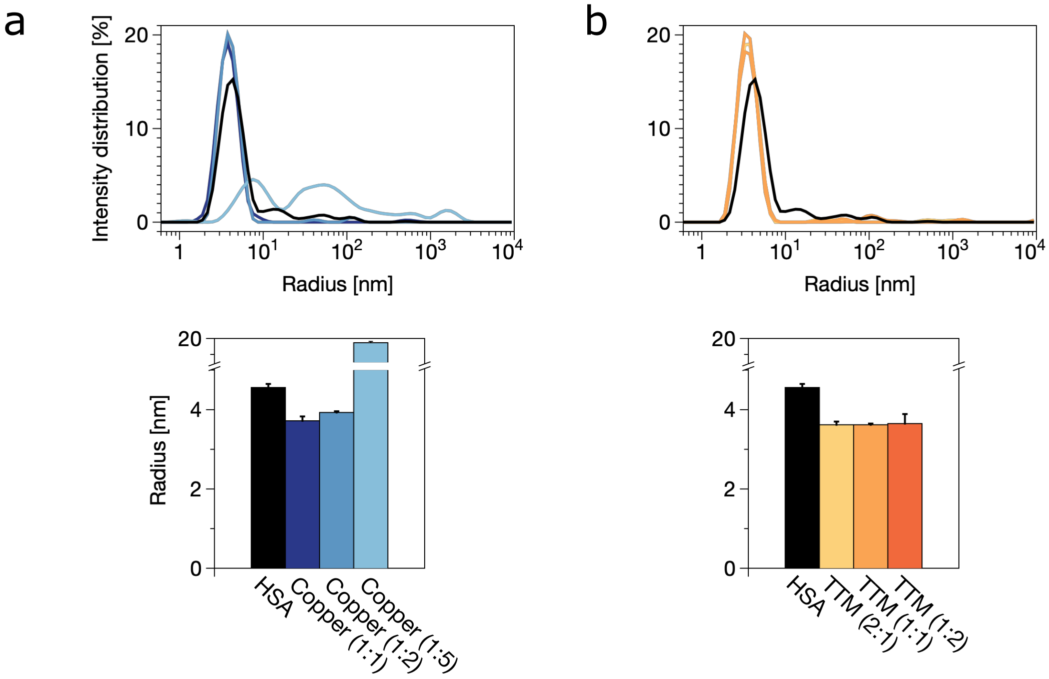

Figure S4. Dynamic light scattering (DLS) confirms structural compaction upon addition of (a) $CuCl_2$, and (b) TTM. At higher $CuCl_2$ concentration (1:5), HSA forms larger aggregates.

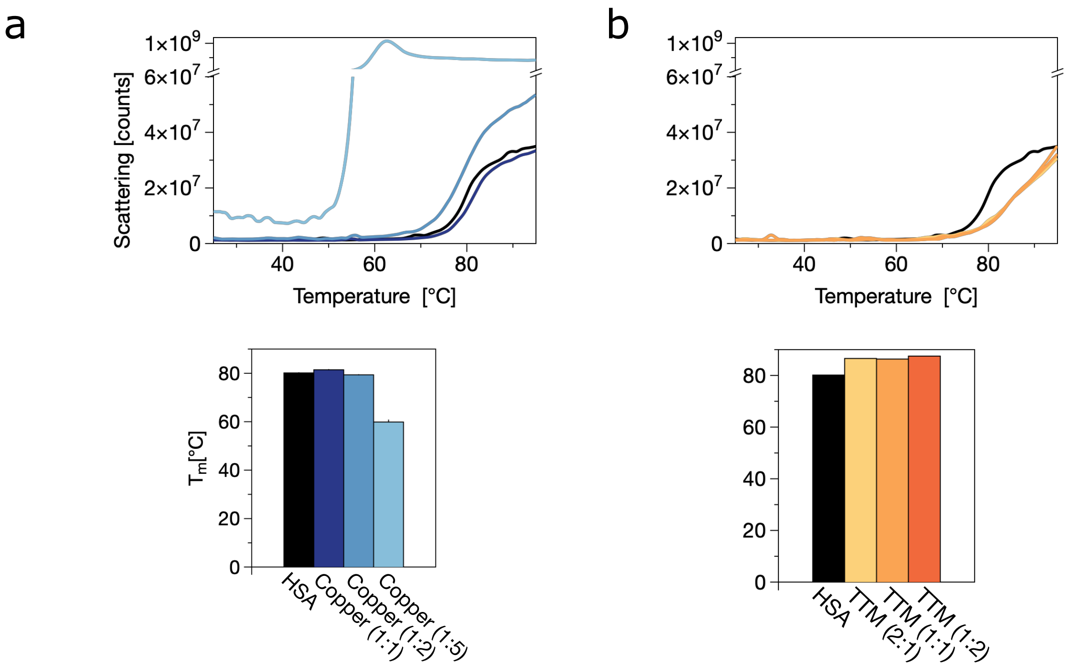

Figure S5. (a) Thermal denaturation profiles reveal that high $CuCl_2$ concentrations destabilise HSA and promote aggregation. (b) TTM confers partial thermal protection against denaturation.

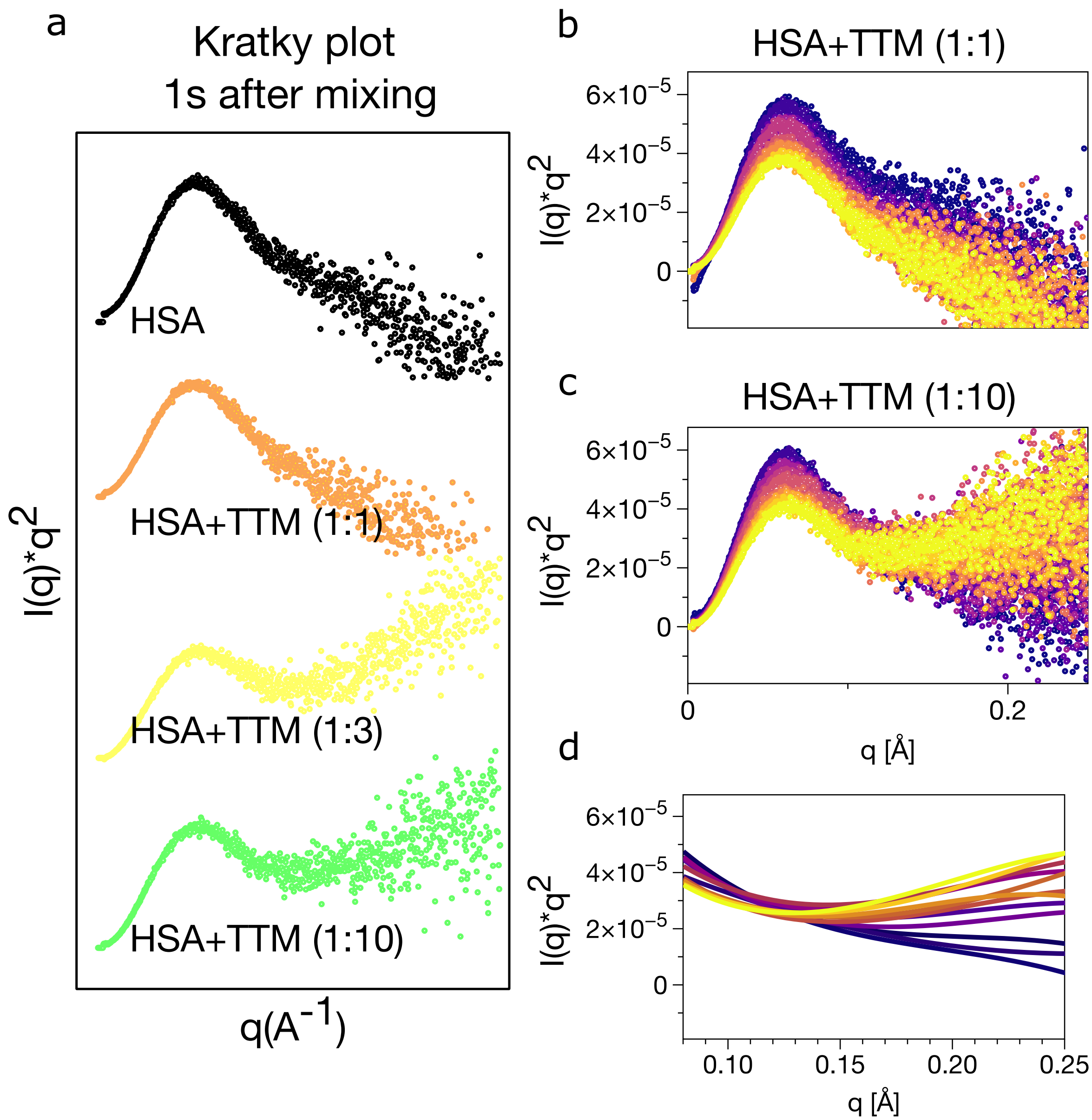

Figure S6. Kratky plots from time-resolved SAXS measurement show that (a) HSA and HSA + TTM (1:1) remain folded, while higher TTM concentration (1:3, 1:10) induce partial unfolding. (b, c) Time evolution of the Kratky plots of 1:1 and 1:10 TTM conditions. (d) Cubic fit of HSA+TTM (1:10) shows increased flexibility in the mid-q region (from blue to yellow).

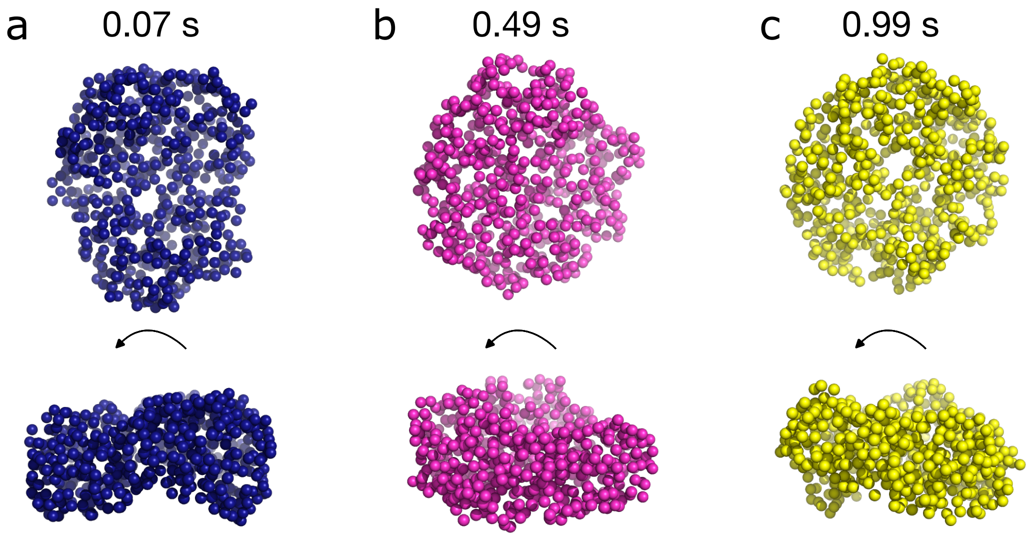

Figure S7. Ab-initio GASBOR models of HSA+TTM (1:10 molar ratio) at (a) 0.07 s (χ^2=1.041), (b) 0.49 s (χ^2=1.243) and (c) 0.99 s (χ^2=1.235) after mixing. All models suggest compaction with time under high TTM conditions.

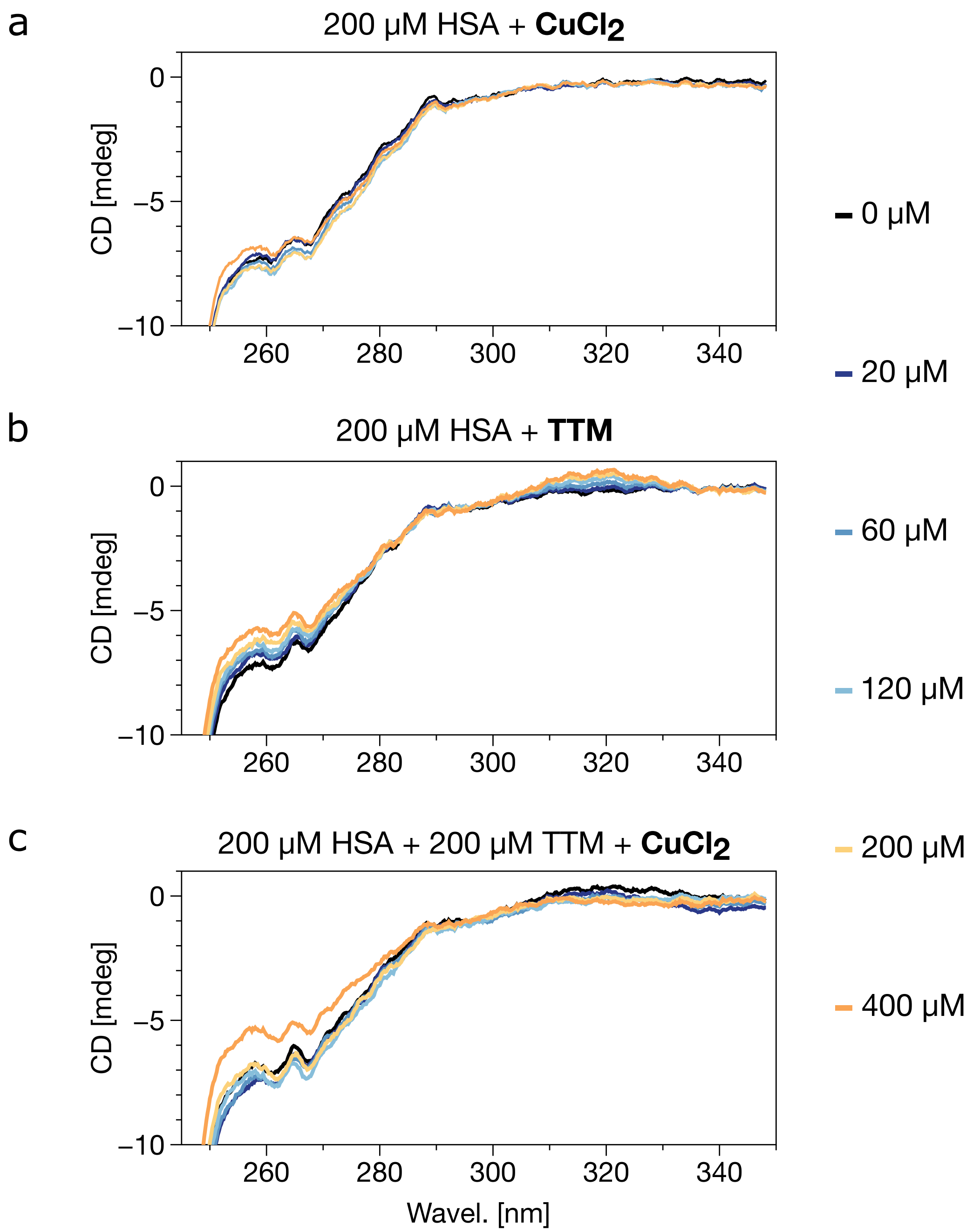

Figure S8. Near-UV CD spectra of 200 µM HSA with titration of (a) $CuCl_2$, (b) TTM and (c) $CuCl_2$ to HSA+TTM mixture. The additive was added to a final concentration of 0, 20, 60, 120, 200 and 400 µM. TTM induces changes in aromatic region and a 320 nm S-S-sensitive signal, which disappears upon Cu addition, indicating modulation of disulphide environment.

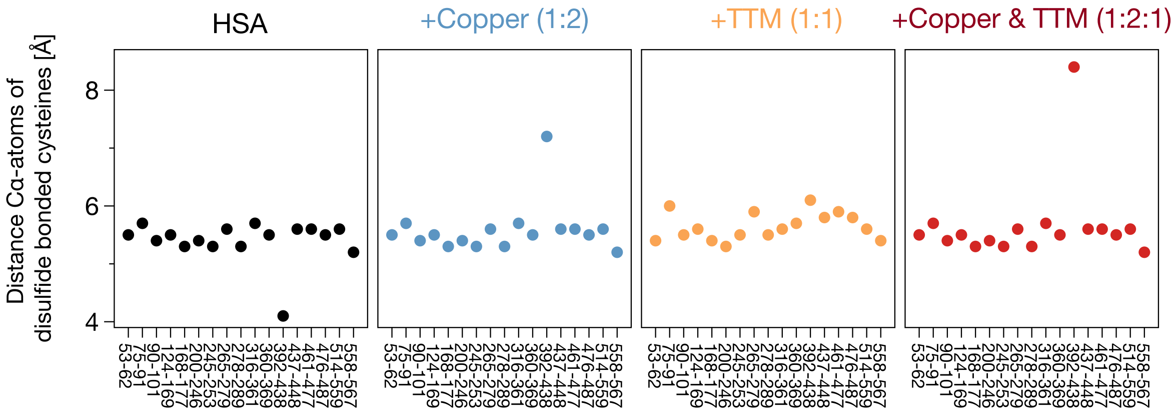

Figure S9. Cα-Cα distances across all 17 disulphide bonds in HSA based on SAXS refined models.

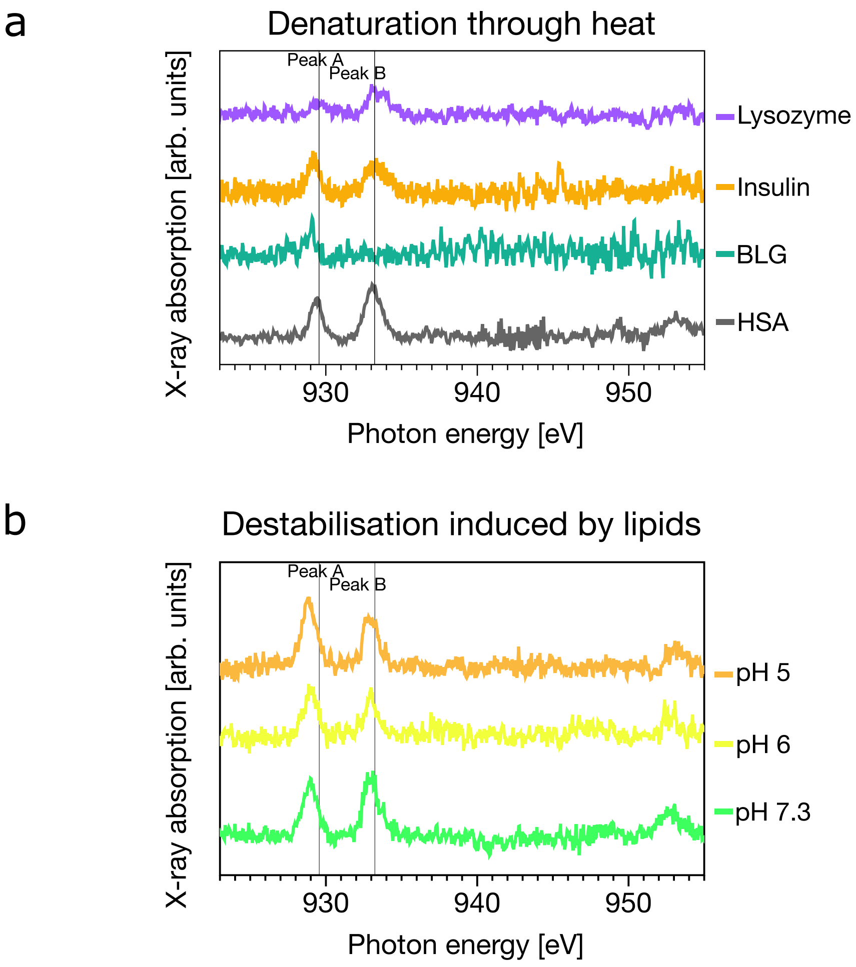

Figure S10. (a) Soft XAS spectra of heat-denatured globular proteins. (b) SOD1 spectra after lipid-induces destabilisation. Denaturation affects copper redox behaviour.

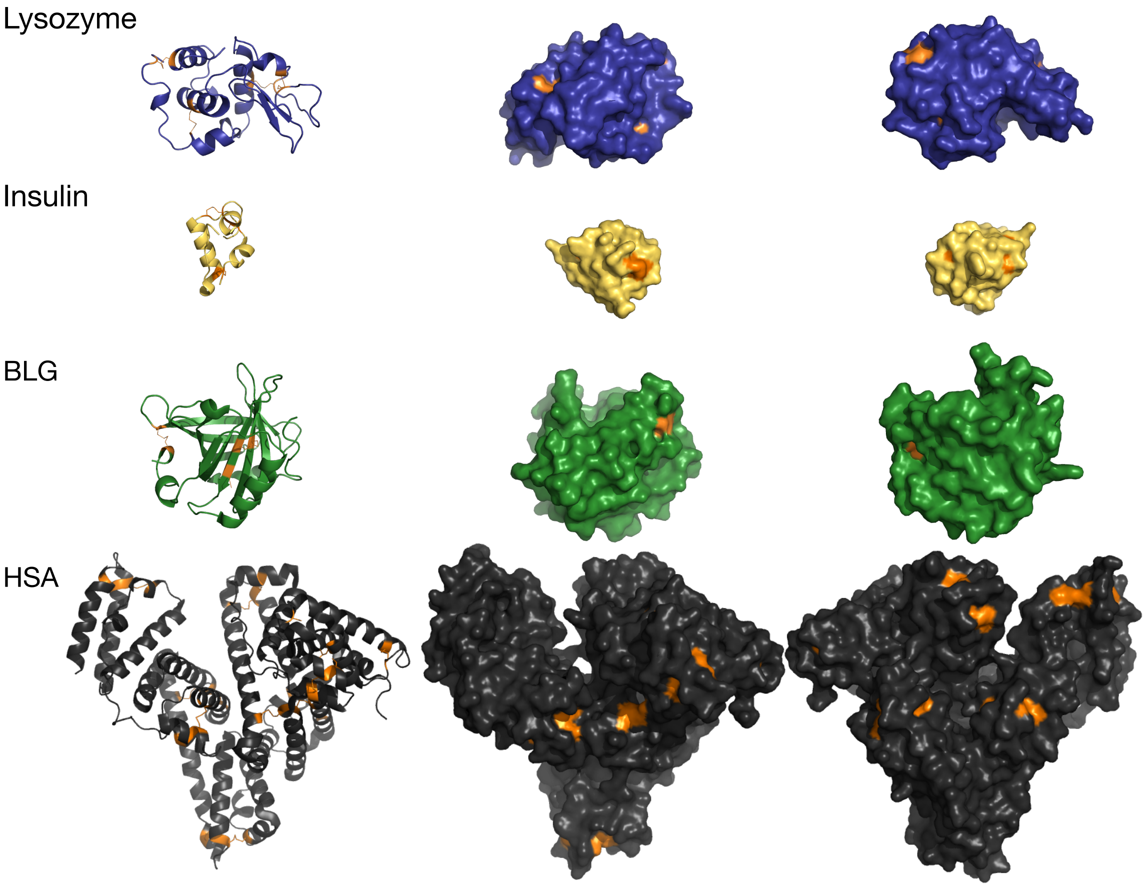

Figure S11. Structural representations of lysozyme (PDB ID: 1REX), insulin (PDB: 3I40), BLG (PDB ID: 3NPO) and HSA (PDB ID: 7WLF) as a cartoon (left) and the surface (middle and right). The Cys residues and CC bonds are highlighted in orange.

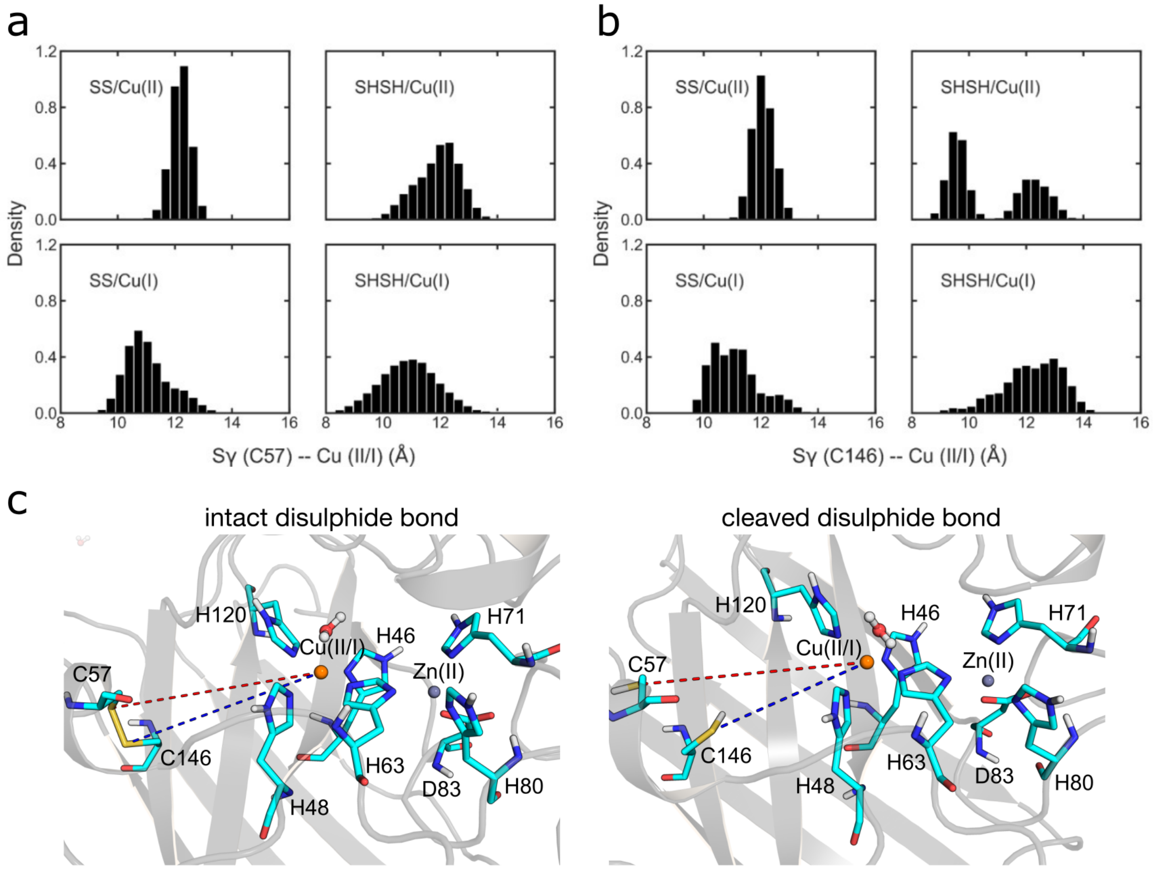

Figure S12. Histograms of Cu (II/I) the distances in SOD1 with (a) Cys57 and (b) Cys146 obtained from the classical MD simulations. SS is referred as a disulphide bond between C57 and C146, while SHSH is referred as no disulphide bond between C57 and C146. (c) QM regions used in QM/MM MD simulations, comparing intact and cleaved Cys57-Cys146 disulphide bond in SOD1.

References

1. Ahl, M., Lindberg, M. J. & Tibell, L. A. Coexpression of yeast copper chaperone (yccs) and cuzn-superoxide dismutases in escherichia coli yields protein with high copper contents. *Protein expression purification* **37**, 311–319 (2004).
2. Piamonteze, C. *et al.* X-treme beamline at sls: X-ray magnetic circular and linear dichroism at high field and low temperature. *J. Synchrotron Radiat.* **19**, 661–674 (2012).
3. Strange, R. W. *et al.* The structure of holo and metal-deficient wild-type human cu, zn superoxide dismutase and its relevance to familial amyotrophic lateral sclerosis. *J. Mol. Biol.* **328**, 877–891 (2003).
4. Jorgensen, W. L., Chandrasekhar, J., Madura, J. D., Impey, R. W. & Klein, M. L. Comparison of simple potential functions for simulating liquid water. *The J. Chem. Phys.* **79**, 926–935 (1983).
5. Li, P. & Merz, K. M. J. Taking into account the ion-induced dipole interaction in the nonbonded model of ions. *J. Chem. Theory Comput.* **10**, 289–297 (2014).
6. Case, D. A. *et al.* Ambertools. *J. Chem. Inf. Model.* **63**, 6183–6191 (2023).
7. Salomon-Ferrer, R., Götz, A. W., Poole, D., Le Grand, S. & Walker, R. C. Routine microsecond molecular dynamics simulations with amber on gpus. 2. explicit solvent particle mesh ewald. *J. Chem. Theory Comput.* **9**, 3878–3888 (2013).
8. Ryckaert, J.-P., Ciccotti, G. & Berendsen, H. J. Numerical integration of the cartesian equations of motion of a system with constraints: molecular dynamics of n-alkanes. *J. Comput. Phys.* **23**, 327–341 (1977).
9. Darden, T., York, D. & Pedersen, L. Particle mesh ewald: An n∘log(n) method for ewald sums in large systems. *The J. Chem. Phys.* **98**, 10089–10092 (1993).
10. Warshel, A. & Levitt, M. Theoretical studies of enzymic reactions: Dielectric, electrostatic and steric stabilization of the carbonium ion in the reaction of lysozyme. *J. Mol. Biol.* **103**, 227–249 (1976).
11. da Silva, A. W. S. & Vranken, W. F. Acpype - antechamber python parser interface. *BMC Res. Notes* **5**, 367 (2012).
12. Abraham, M. J. *et al.* Gromacs: High performance molecular simulations through multi-level parallelism from laptops to supercomputers. *SoftwareX* **1**, 19–25 (2015).
13. Cp2k (2022).
14. Kühne, T. D. *et al.* CP2K: An electronic structure and molecular dynamics software package - Quickstep: Efficient and accurate electronic structure calculations. *J. Chem. Phys.* **152**, 194103, DOI: 10.1063/5.0007045 (2020).
15. VandeVondele, J. *et al.* QUICKSTEP: Fast and accurate density functional calculations using a mixed gaussian and plane waves approach. *Comput. Phys. Commun.* **167**, 103–128, DOI: 10.1016/j.cpc.2004.12.014 (2005).
16. Goedecker, S., Teter, M. & Hutter, J. Separable dual-space gaussian pseudopotentials. *Phys. Rev. B* **54**, 1703–1710, DOI: 10.1103/physrevb.54.1703 (1996).
17. Krack, M. Pseudopotentials for h to kr optimized for gradient-corrected exchange-correlation functionals. *Theor. Chem. Acc.* **114**, 145–152, DOI: 10.1007/s00214-005-0655-y (2005).
18. Blanchet, C. E. *et al.* Versatile sample environments and automation for biological solution x-ray scattering experiments at the p12 beamline (petra iii, desy). *J. applied crystallography* **48**, 431–443 (2015).
19. Wang, Z.-m. *et al.* Structural studies of several clinically important oncology drugs in complex with human serum albumin. *Biochimica et Biophys. Acta (BBA)-General Subj.* **1830**, 5356–5374 (2013).
